# Evaluation and Uptake of an Online ADHD Psychoeducation Training for Primary Care Health Care Professionals: Implementation Study

**DOI:** 10.2196/59365

**Published:** 2025-07-11

**Authors:** Blandine French, Hannah Wright, David Daley, Elvira Perez Vallejos, Kapil Sayal, Charlotte L Hall

**Affiliations:** 1 Institute of Mental Health, Jubilee Campus, University of Nottingham Innovation Park, Triumph Road, Nottingham, NG7 2TU, United Kingdom, 44 0115 951 5151; 2 Applied Psychological Practice, University of Nottingham Innovation Park, Nottingham, United Kingdom

**Keywords:** ADHD, online training, general practice, psychoeducation, training, implementation report, primary care, primary care health professionals, healthcare professionals, survey, feedback, training program, neurodevelopmental conditions

## Abstract

**Background:**

Health care professionals seldom receive training on neurodevelopmental conditions such as attention-deficit/hyperactivity disorder (ADHD). An online training was co-developed to address some of the gaps in knowledge and understanding in primary care. A randomized controlled trial demonstrated that the training increased knowledge and confidence and improved practice.

**Objective:**

This report highlights the implementation of the training in practice and follow-up 4 years post evaluation.

**Methods:**

The online ADHD training comprises 2 modules: “Understanding ADHD” and “The Role of the GP,” each taking approximately 45 minutes to complete. The training targets general practitioners primarily but is open to other health care professionals and parents. Feedback was collected through a survey at the end of the training, and the training has been widely adopted by various organizations internationally and nationally.

**Results:**

Between December 2019 and January 2024, the “Understanding ADHD” module was accessed more than 13,486 times, while the “Role of the GP” module was accessed 7018 times, primarily by users from the United States and the United Kingdom. Survey results from both modules showed positive feedback with high ratings for usefulness, likelihood to inform practice, and recommendation to colleagues. Some suggestions for improvement included reducing the negative focus on ADHD consequences and incorporating more positive aspects of ADHD.

**Conclusions:**

This ADHD online training program, despite facing implementation challenges, has seen positive outcomes, including international translation and high user ratings. Suggestions for improvement were received, but some were not feasible due to regional variations in ADHD pathways. The training’s impact extended beyond GPs to other health care professionals, although the COVID-19 pandemic posed obstacles to dissemination efforts. Nonetheless, ongoing plans aim to expand the training’s implementation globally.

## Introduction

### Background

Attention-Deficit Hyperactivity Disorder (ADHD) is a neurodevelopmental condition characterized by symptoms of persistent inattention or hyperactivity-impulsivity, which causes clinical impairment in academic and social functioning [1] affecting approximately 5% of children [2] and 2.5% of adults [3]. Having ADHD carries many additional risks [4], and these are worsened when unsupported and undiagnosed [5], often leading to an increase in access to primary care services [6].

In the United Kingdom, access to care for children and adults with attention-deficit hyperactivity disorder (ADHD) is complex, starting from general practitioners (GPs) in primary care or schools who refer on to specialist services for diagnosis and treatment (psychiatry, Children and Adolescent Mental Health Services, etc). Health care professionals, including GPs, receive little or no training on ADHD. This significantly impacts access to care for many children and adults as GPs are the main gatekeepers for specialist services [7,8]. To fill this gap in ADHD training, an online training was coproduced with GPs to improve GPs’ knowledge about ADHD [9]. The stepwise, coproduction approach toward developing this online ADHD training for GPs began with a preparatory workshop in order to highlight the relevant topics to be included in the intervention, from which educational videos were then developed, as well as the content and format for the training. Two workshops were then conducted with GPs, leading to further refinement of the video content and subsequently the final intervention. A pilot usability study (N=10 GPs) was conducted to assess the intervention’s acceptability, feasibility, and accessibility. The online training included interactive psychoeducation elements reinforced with activities and videos lasting a total of 45 minutes. The content included enough information for GPs to identify ADHD and better understand the condition. The resulting intervention was then evaluated through a randomized controlled trial (RCT) [10] in GP practices based in England where 221 GPs took part. The evaluation of this training demonstrated that GPs’ knowledge and confidence significantly improved, misconceptions decreased, and attitudes and reported practice changed [10]. The unique aspect of this training lies in the strong coproduction element, with GPs being involved throughout the development and review process and within the evaluation through an RCT, which is rarely done for education packages. The coproduction element, reviews, and evaluation aspect of the original project took over 2 years from January 2018 to March 2020. To our knowledge, no other online training package has been developed and evaluated for ADHD in primary care in the United Kingdom.

The original evaluation of this training terminated in March 2020. Since then, we have spent time implementing the training in GP practices, in alignment with the British National Institute for Healthcare Research (NIHR) priority settings in digital support for primary care [11].

This publication describes the impact, engagement, and implementation of the original ADHD training program, 4 years beyond completion of data collection.

### Aims and Objectives

This project aimed to implement and evaluate the ADHD online training into practice, beyond the scope of the original project. The implementation and impact of the training are measured through website access analytics and responses to survey questions.

## Methods

### Blueprint Summary and Technical Design

**Figure 1.**
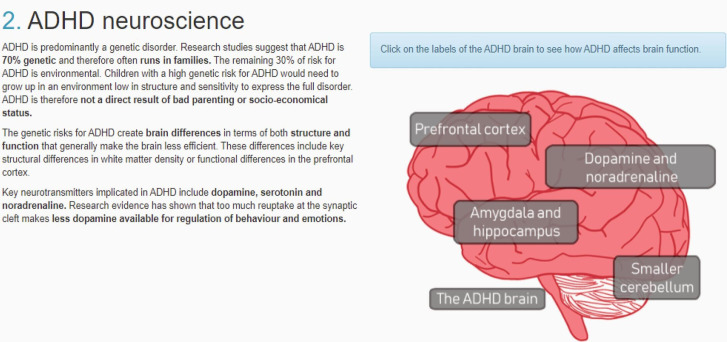
Example of educational module page from the training. ADHD: attention-deficit/hyperactivity disorder.

The online ADHD training is a psychoeducation program consisting of 2 modules, one on “Understanding ADHD” and one on “the role of the GP in the care pathway.” The training takes approximately 45 minutes to complete and can be accessed freely online (link in [12]; example of an education module page in Figure 1).

The training is hosted on a secure platform within the University of Nottingham Health E-learning and Media team (HELM). It has been developed to be easily accessible on a computer or a mobile device. Further details on the original project can be found online [13].

### Target

The training is principally aimed at general practitioners or other medical doctors. However, while the module “Role of the GP” in the care pathway focuses on primary care, the module “Understanding ADHD” can be useful for anyone including other health care professionals, education professionals, and parents. Additionally, although the training has elements of the care pathway that are specific to the United Kingdom, a large proportion of the training is relevant to other countries and settings as well (psychoeducation on ADHD, effect of medication, types of interventions, etc) and therefore was found to be useful in different contexts. The original RCT had specific inclusion criteria, but for this aspect of implementation, no restrictions were implemented on either module. The online training is freely available and hosted on a university server that is widely secure and accessible, including from health care servers (eg, SystmOne). This was essential as a lot of health care servers can block external links.

### Survey

The survey was developed by the lead researchers. Following a pragmatic approach prioritizing quick time response, only 4 questions were presented to participants in the training. As the training is freely available and no longer part of a study, the questions were optional and presented upon completion of each module. Not all participants took part in both modules, and we wanted to capture the values of each. While the survey is aimed primarily at GPs, anyone could take part in the module, and we anticipated that many other health care professionals would take part in this training. Therefore, the questions were targeted to capture clinical impact on practice with health care, in line with the initial aim of the training. In addition to 3 demographic questions (age, occupation, and gender), 4 evaluation questions were presented:

1. How useful did you find the information in this program?
2. How likely is this information going to inform your practice?
3. Would you recommend this training to your colleagues?
4. Any other comments on the intervention?

Participants were asked to select a score on a scale of 1‐10 (1: not at all and 10: extremely) to represent how much they agreed with the evaluation questions.

### Data

The data and analytics generated from the survey are stored online within the HELM platform and only accessible by the HELM team. The feedback questionnaire was anonymous and voluntary for anyone taking part in the training.

### Participating Entities and Dissemination

Many partner organizations have adopted, distributed, and implemented the training over the last 4 years. These include the Royal College of General Practice (RCGP), ADHD Europe, the Association for Child and Adolescent Mental Health (ACAMH), the Academic Health Science Network (AHSN), local GP training hubs, European ADHD research networks (EUNETHYDIS), the University of Montpellier, The University of Dublin and the ADHD collective.

Presentations about the training have been delivered by the lead researcher to groups locally, nationally (eg RCGP) and internationally (eg ADHD Europe). The training has been accredited by the RCGP, the leading professional organization for GP training and accreditation in the United Kingdom, as part of its Continuing Professional Development (CPD) program. Accreditation included further peer review from GPs and refinement of text in line with national and international guidelines (National Institute for Health and Care Excellence and *Diagnostic and Statistical Manual of Mental Disorders, Fifth Edition*). Internationally, the training has been translated into 3 languages (German, French, and Spanish), and ongoing collaboration with leading European ADHD research networks (EUNETHYDIS) has started to develop evaluation and implementation of the translated versions.

The training development and initial RCT evaluation were funded by the Economic and Social Research Council (ESRC) through a doctorate training program. The training also received a non-profit grant from Takeda (a pharmaceutical company) to support the online development and trial.

### Sustainability and Budget

The original project funds from Takeda allowed for the intervention to be developed and hosted on the free accessible HELM platform. A booster grant from the same funders also allowed for the translations into other languages to be completed, but aside from these, no other budget was available for the long-term implementation of the training. As a university employee, the lead researcher has driven the implementation in her own time by giving workshops, training, and presentations to specific groups over the last few years.

### Ethical Considerations

Ethics approval for the RCT and the ongoing evaluation was received from the University of Nottingham, Faculty of Medicine Research Ethics Committee (reference 19/HRA/1028) and from the Nottinghamshire Healthcare National Health Service (NHS) Foundation Trust Research and Development department (project ID 257567). Participants in the survey consented to their data being used. In accordance with ethical standards with IRB and with the Helsinki Declaration of 1975, as revised in 2000. Participants were not compensated for their time in providing feedback. All responses were anonymous and unidentifiable.

## Results

### Coverage

**Figure 2.**
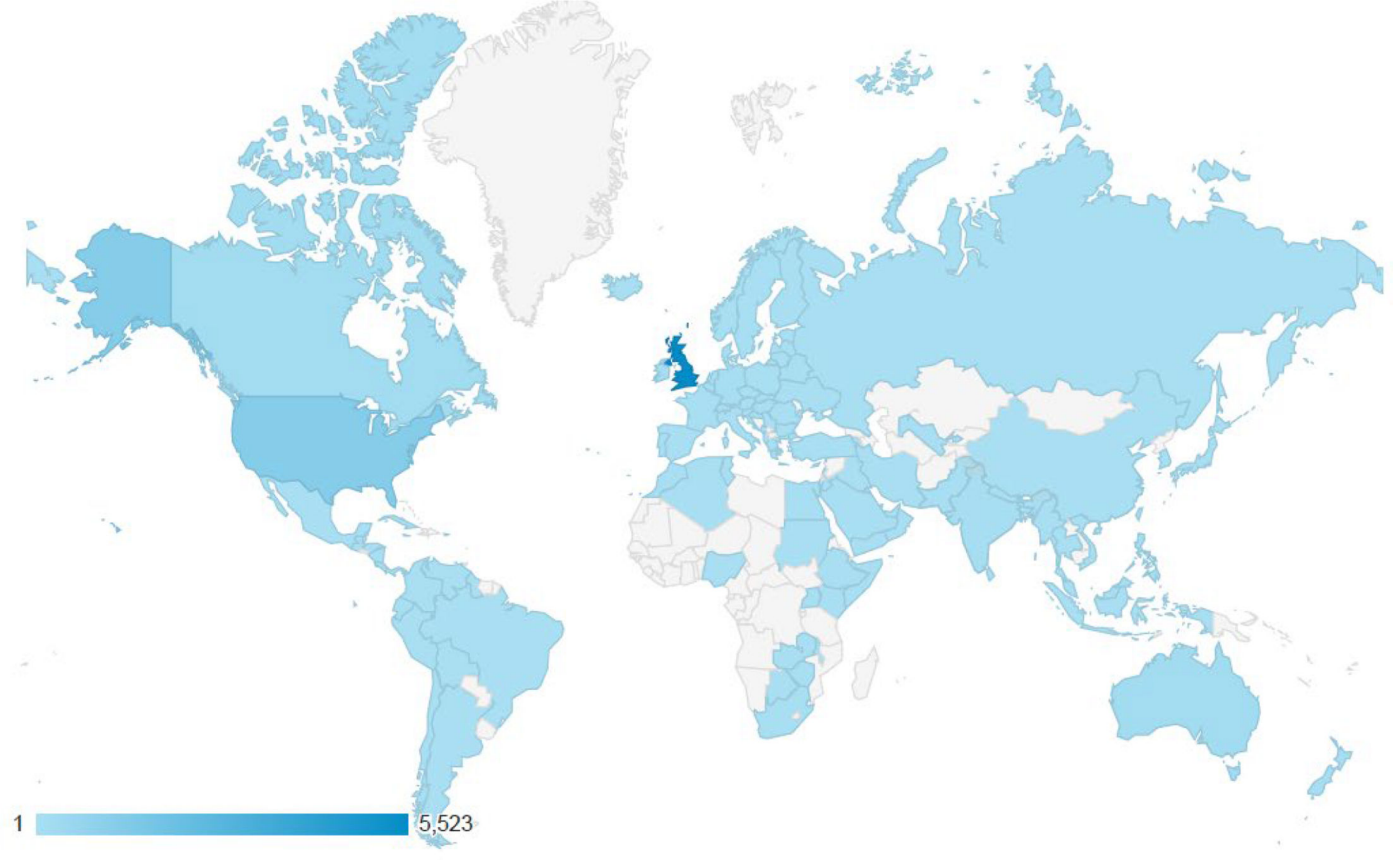
Countries that have accessed the attention-deficit/hyperactivity disorder (ADHD) training from 2019 to 2024.

Between December 2019 and January 2024, the “Understanding ADHD” module was accessed more than 13,486 times with 1484 (11%) of users returning visitors. Many partner organizations have adopted and disseminated the training. Most users were from the United States and the United Kingdom (33% and 57%, respectively). The “role of the GP in the care pathway” module was accessed 7,018 times with 716 (10.2%) of returning visitors. Most users were from the United States and the United Kingdom (20% and 76%, respectively), but the training was also accessed by users based in another 120 countries (Figure 2).

### Outcomes: Survey Results

#### “Understanding ADHD” Module

A total of 648 participants responded (134 males, 496 females, and 18 unknown), with a mean age of 40.4 (SD 11.32) years (range 16‐81 years). On average, participants rated the information in the resource 8.47 (SD 1.93) for usefulness and 8.26 (SD 2.11) for how likely this information was going to inform their practice. A total of 611 of 631 (96.8%) participants would recommend the training resource to their colleagues. Table 1 represents the demographics and responses given, separated into different occupations.

**Table 1.** Demographic data and average evaluation questionnaire responses for the “Understanding ADHD^a^” module, separated by occupation category.

Variables	Occupation				
	GP^b^(n=66)	Other health care professionals(n=285)	Mental health professionals(n=91)	Health care students(n=47)	Other occupations^c^(n=159)
Gender					
Men	22	74	13	4	21
Women	43	209	76	41	127
Unknown gender	1	2	2	2	11
Age (years), mean (SD)^d^	44.97 (11.74)	40.89 (11.30)	40.01 (11.56)	31.85 (9.20)	40.57 (12.83)
Usefulness rating^e^,mean (SD)	8.36 (1.89)	8.61 (1.83)	8.69 (1.62)	8.39 (1.92)	8.13 (2.20)
Likelihood that information would inform practice^e^,mean (SD)	8.30 (1.83)	8.49 (1.95)	8.64 (1.66)	8.40 (2.05)	7.56 (2.55)
% Respondents that would recommend the training resource to their colleagues	96.97	96.74	100.00	97.87	94.74

a ADHD: attention-deficit/hyperactivity disorder.

b GP: general practitioner.

c Other occupations include non–health care students, homemakers, project managers, teachers, and teaching assistants.

d Data taken only from those who responded. Overall, 13 did not respond to age.

e On a scale of 1‐10, 1 being not at all, and 10 being extremely.

##### Positive Feedback

The free-text response box yielded 117 responses about the resource, 104 (89%) of which were positive. Participants felt that the resource was comprehensive and clear and appreciated the range of mediums used, including sound bites, videos, text, and interactive quizzes. Multiple comments acknowledged the benefit of including personal lived experiences to help solidify the information included.

Many commented that the resource will be valuable to them in their practice as a GP or healthcare professional, eg, “I am better informed and ready to be more useful to my patients,” and others said it was helpful to them on a more personal level (eg, understanding their children or colleagues with ADHD).

This intervention is amazing and the value availed of by the public, ADHDers, teachers and medical professionals cannot be underestimated in fact I would say it is immeasurable! [Homemaker]

Fantastic resource. Thank you so much. The simple and accessible explanations are brilliant. [Researcher]

Easy to read, absorb and navigate. Highly informative educational resource, one of the best I have come across as an individual who suffers from ADHD, thank you! [Carer]

##### Suggestions for Improvement

The most common critical feedback was that the module focused too heavily on the negative consequences and risks associated with ADHD. Participants expressed that there are positives associated with ADHD that could be mentioned and that people with ADHD can still be successful, employed, and intelligent. Some highlighted concern that the slides on the problems with ADHD “played into the stigma that often stops people from being accepted for an assessment in the NHS.”

It would also be good to emphasise the potential strengths in individuals with ADHD for example creativity and the benefits of hyper-focus. [Clinical psychologist]

Another issue was the lack of representation in the videos for non-White individuals, and therefore more diversity in ethnicity was requested. A few comments suggested the need for an explicit reference to the differential presentation and diagnosis rates between males and females.

### “Role of the GP” Module

A total of 308 participants responded to this module feedback questionnaire (64 males, 241 females, and 3 unknown), with a mean age of 39.9 (SD 11.15) years (range 18‐67 years). On average (mean), participant rating was 8.12 (SD 2.16) for usefulness, and 7.98 (SD 2.14) on how likely this information is going to inform your practice, demonstrating that overall participants responded positively, finding the program to be useful for their knowledge and improving their practice. Additionally, 291 of 299 respondents (97.3%) would recommend the training resource to their colleagues.

Table 2 shows the age, gender, and answers, separated by the participant’s professional group. The ratings appear to be quite similar between each occupation group. The GP group gave the lowest rating for usefulness, likelihood to inform practice, and likelihood to recommend to colleagues, whereas mental health professionals gave the highest.

**Table 2.** Demographic data and average evaluation questionnaire responses for the “Role of the GP” module, separated by occupation category.

Variables	Occupation				
	GP^a^(n=66)	Other health care professionals(n=128)	Mental health professionals(n=30)	Health care students(n=30)	Other occupations^b^(n=54)
Gender					
Men	19	30	6	4	5
Women	46	98	24	26	47
Unknown gender	1	0	0	0	2
Age (years), mean (SD)^c^	45.40 (9.59)	39.00 (10.27)	39.40 (11.15)	31.50 (12.35)	39.67 (12.42)
Usefulness rating^d^,mean (SD)	7.92 (2.16)	8.02 (2.02)	8.67 (1.60)	8.40 (2.04)	8.15 (2.30)
Likelihood that information would inform practice^d^,mean (SD)	7.74 (2.18)	7.93 (2.09)	8.53 (1.67)	8.50 (2.01)	7.78 (2.39)
% Respondents that would recommend the training resource to their colleagues	93.80	98.40	100.00	96.60	98.08

a GP: general practitioner.

b Other occupations include non–health care students, a local government manager, a homemaker, and a teaching assistant.

c Data taken only from those who responded. Overall, 4 did not respond to age.

d On a scale of 1‐10, 1 being not at all, and 10 being extremely.

#### Positive Feedback

The free-text response box yielded 75 responses about this resource, 61 of which were positive (81%). Many comments said that the resource was “very interesting,” “informative,” and “useful.” There was also praise regarding the clarity and presentation of the information, which facilitated easy understanding. Some respondents also commented that it was particularly insightful to include a video from the point of view of a GP discussing their own difficulties in getting a diagnosis, adding a unique and important perspective. These positive comments came from participants with a range of backgrounds demonstrating the benefits of this resource for GPs as well as other professions.

Clear and well presented. So many people complicate ADHD and so it becomes difficult to learn - this was a perfect way to introduce it - and the GP with ADHD was a fantastic resource. [GP]

Very detailed, lots of information and suitable diagrams and videos of experiences also helped. [Student Mental Health Nurse]

This is a really good teaching resource. I will be recommending that all new nursing staff also have access to this. [Clinical Specialist ADHD Nurse]

#### Suggestions for Improvement

A few of the free-text responses gave some suggestions for other things to include in this module. A couple of participants asked for more information about the assessment process and specific information about the role of the GP in shared care, with the roles of nurses and support workers. There was a suggestion from a few GPs to include free resource signposting for them to give to patients and parents to help with management.

It could do with more detail and clinical scenarios. Also important is the association and differential diagnosis of other comorbidities, addiction/personality disorders/anxiety depression. That may indeed be beyond the scope of this website. [GP]

As these participants acknowledged themselves, this module focused on the role of the GP in ADHD, so did not aim to include comorbid diagnoses.

Another participant gave a specific suggestion for the “What is ADHD” section to include clearer differentiation about the presentation in girls and boys or women and men and more detail on how ADHD impacts mental health when it is not diagnosed or well-treated.

A care assistant expressed their frustration about the care system in their part of the United Kingdom in referring to the Association for Child and Adolescent Mental Health or adult mental health services for ADHD, commenting that “Your information makes going to the doctors and getting a diagnosis seem easy.”

Finally, a couple of other respondents stated that this resource did not add anything new to their knowledge and would not change their practice as a GP, although this may not be reflective of the quality of the resource, but of this participant’s existing knowledge being sufficient or extensive.

## Discussion

### Principal Findings

Overall, the implementation of this funding has been very positive, particularly in the light of no secured economic or staff resources to support this. The program has been translated internationally, reaching more than 120 countries and has been translated into 3 different languages. The feedback has been predominantly positive, and the intervention has received consistently high ratings regarding its acceptability and usefulness.

### Lessons Learned

Many lessons were learned from the feedback as well as from the dissemination and implementation process.

The feedback highlighted some limitations about how the training had been framed, the lack of representation, and gender differences. The coproduction aspect of this training aimed to maximize the accessibility and usability of the training by GPs. Service users also reviewed the training before its dissemination, but their input was not as significant as that from GPs. Some of these issues might have been avoided by more thorough service-user involvement. Striking the balance between the different stakeholders was complex as, in this instance, their input at times was not compatible.

The dissemination of the training in practice within the United Kingdom was also very complex. In the United Kingdom, there is no single training organization that can adopt training and trickle down to all GPs in a top-down manner. Each area of the United Kingdom has separate training programs that are decided at a regional level, and linking with all different regions is difficult. Therefore, the implementation had to be conducted in a bottom-up manner, which is time-consuming and has limited reach. We were also unable to reliably report data pertaining to user engagement, such as completion rate and number of visits. These numbers were biased by the ongoing use of the tools as a training module, and therefore the numbers do not represent realistic interactions.

It is also important to note that the dissemination of the training started in March 2020. In many countries including the United Kingdom, this was the beginning of the COVID-19 pandemic. This would have had significant impacts on the implementation of the training. First, GPs' priorities had to change very quickly, and practices adapted in a very stressful environment. Dealing with the direct consequences of the pandemic became the priority, with taking part in CPD or a better understanding of ADHD becoming less important. Although the demand for confinement significantly increased ADHD symptoms expressions and associated impairment for some [14] and an increase in referrals postpandemic [15], it was difficult for GPs to prioritize this issue, as is demonstrated by the significant decrease in referrals during lockdowns [16]. Second, the research team was not able to actively reach out, disseminate, and give presentations for a long time, which also impacted the implementation of the training in practice.

Finally, the implementation process has not been without challenges. The UK system for training health care professionals is not easy to navigate. Outreach events have been useful in engaging stakeholders and generating uptake in the training; however, this led to only pockets of health care professionals being trained and did not extend wider than the reach for the event. The aim of the evaluation was to be pragmatic so that we could evaluate the implementation in routine primary care settings; for this reason, the evaluation questions were simple, and free text comments were only provided by a minority of users.

Some suggestions for improvement have been raised, which will be considered in alterations to the training, but it is important to note that these were from a minority of users (30/9000). Additionally, some of the suggestions were not possible to implement, for example, in local pathways and national practices. The local pathways for ADHD vary widely between regions and countries. They also often change regularly, and information quickly becomes outdated [17-19]. Therefore, while the information on pathways would be very beneficial, it is impossible to capture all regional and national variations.

While the training was primarily aimed at GPs, it was always clear that it would benefit many other health care professions working alongside primary care. By gaining a better understanding of the GP’s role, the roles of other professionals will become clearer. Therefore, it is very positive that so many other health care professionals accessed the training and gained a better understanding of ADHD, significantly widening the impact and reach of the resource.

In conclusion, this coproduced and evidence-based training shows ongoing benefits, acceptability, and usefulness in practice. The results from this implementation demonstrated a wider use to other health care professionals and international reach. Ongoing implementation plans aim to support further the wider implementation of this training, principally in other countries.

## Abbreviations

ADHD: attention-deficit/hyperactivity disorder
CPD: Continuing Professional Development
GP: general practitioners
RCT: randomized controlled trial
